# Additional interventions for enhancing the quality-of-life of older adults using hearing aids: a systematic review and narrative synthesis

**DOI:** 10.1007/s11136-026-04219-7

**Published:** 2026-03-13

**Authors:** Minji Kang, Yunji Han, Hyejoon Kim

**Affiliations:** 1 Seoul National University College of Nursing and Research Institute of Nursing Science, 103 Daehak-ro, Jongno-gu, Seoul, 03080 Republic of Korea; 2 Center for World-Leading Human Care Nurse Leaders for the Future By Brain Korea 21 (BK 21) Four Project, College of Nursing, Seoul National University, Seoul, Republic of Korea

**Keywords:** Hearing aids, Interventions, Hearing loss, Older adults, Quality of life

## Abstract

**Purpose:**

Hearing loss in older adults substantially affects quality of life. Although hearing aids improve hearing, their effects vary. Combining hearing aids with additional interventions may offer added benefits, yet it remains unclear which quality-of-life domains benefit most. This systematic review examined the effects of hearing aids plus supplementary interventions, compared with hearing aids alone, on quality-of-life outcomes.

**Methods:**

Randomized controlled trials (1996–2024) were identified from seven databases. Eligible studies included adults aged ≥ 65 years and compared hearing aids combined with additional interventions with hearing aids alone. Outcomes were categorized into physical, psychological, and older-adult-specific domains using the WHOQOL-100 and WHOQOL-OLD frameworks. Risk of bias was assessed using RoB 2, and certainty of evidence using GRADE. A narrative synthesis was conducted.

**Results:**

Twelve randomized controlled trials involving 892 participants were included. Additional interventions comprised individual at-home training, group-based auditory rehabilitation, empowerment strategy, and telehealth. Individual at-home training showed partial benefits in sensory abilities, self-efficacy, and autonomy. Group-based auditory rehabilitation enhanced communication strategies and positive emotional outcomes in some studies. Empowerment strategy showed favorable psychological effects, while telephone-based telehealth interventions demonstrated limited impact on quality of life.

**Conclusions:**

Combining hearing aids with additional interventions may improve psychological and autonomy-related aspects of quality of life in older adults. However, heterogeneity in intervention content and outcome measurement limits firm conclusions. Future studies should use adequately powered randomized controlled trials, standardized outcome measures, and longer follow-up, and WHOQOL guided evaluation to support more comprehensive and comparable evidence.

**Registration:**

No. 605082.

**Supplementary Information:**

The online version contains supplementary material available at 10.1007/s11136-026-04219-7.

## Plain English summary

Hearing loss is common among adults aged ≥ 65 and can substantially diminish quality of life. Many individuals use hearing aids, but their benefit varies and often remains limited. This situation creates a need to understand whether adding supportive interventions to hearing aid use can provide broader improvements in daily functioning and well-being. The key issue addressed in this manuscript is the lack of clear evidence on which aspects of quality of life respond most favorably to combined interventions compared with hearing aids alone. This study aimed to systematically review randomized controlled trials that evaluated hearing aid use combined with supplemental interventions (e.g., auditory training, group-based rehabilitation, empowerment programs, or telehealth), compared with hearing aid use alone. The review found that combined approaches may improve psychological well-being, self-efficacy, and autonomy for some older adults. However, the overall certainty of evidence was low due to variability in study design, intervention content, and outcome measures. These results suggest that multifaceted rehabilitation may offer added value, while highlighting the need for more rigorous and standardized research.

## Introduction

Over 42% of individuals aged 60 years and above globally experience hearing impairment, with age-related hearing loss being the third leading cause of disability [1]. This condition often leads to communication challenges, isolation, frustration, and depressive symptoms, significantly affecting overall quality of life [2–4]. Consequently, it impacts both emotional well-being and participation in social, educational, and professional settings. Research has also indicated that older adults with hearing loss are at increased risk of developing cognitive decline compared with those without hearing loss [5]. Since hearing loss profoundly affects multiple aspects of life in older adults, it is essential to examine the quality of life of those who experience hearing loss throughout their lives.

Quality of life is a complex concept that includes physical health, mental well-being, autonomy, social relationships, and interactions with the environment [6]. The World Health Organization (WHO) defines quality of life as "individuals' perceptions of their position in life in the context of the culture and value systems in which they live and in relation to their goals, expectations, standards, and concerns” [6]. In the WHOQOL framework, quality of life is conceptualized as a multidimensional construct encompassing physical capacity, psychological, level of independence, social relationships, environment, spirituality/religion/personal beliefs. In this context, examining quality of life in older adults with hearing loss could provide healthcare professionals with a more holistic perspective, encouraging them to become more attuned to patients' needs and ultimately fostering the development of interventions that consider the impact on patients' overall well-being.

Hearing aids have been the most common intervention for older adults with hearing loss [7]. A robust body of evidence from systematic reviews indicates that hearing aids improve quality of life [7, 8], but their adoption rate worldwide remains low at approximately 11% [9]. To this end, several nonaudiological factors, such as group instructions and encouragement from others, were found to influence the acceleration of the use of hearing devices, along with audiological factors [10]. Evidence also suggests that hearing aids alone may not be enough to fulfill what people with hearing loss want, as amplification often fails to optimize communication in daily life [11].

Many studies have suggested that combining hearing aids with additional interventions—such as inducing self-affirmation, providing counseling, and educating communication strategies—provides greater benefits for populations with hearing loss and enhances their overall quality of life [12–14]. While some studies have examined the quality of life of older adults with hearing aids, they have focused primarily on health-related quality of life [15] or assessed a single additional intervention without comparison [16]. No studies have examined and synthesized results on which aspects of quality of life benefit more from combining hearing aids with additional interventions than from using hearing aids alone.

Given the lack of comprehensive comparative analyses, this systematic review seeks to synthesize the results and compare the effects of hearing aids combined with additional interventions with those of hearing aids alone on improving the quality of life of older adults with hearing loss.

## Methods

This review followed the PRISMA 2020 guidelines for systematic reviews [17]. A protocol was registered with PROSPERO (No. 605082) prior to the review. Although the study was conducted as a systematic review, heterogeneity in interventions and outcomes precluded meta-analysis, and findings were synthesized narratively. During title revision, the PROSPERO record was withdrawn after being identified by the platform as a scoping review, which is not eligible for registration.

### Eligibility criteria

**Fig. 1:**
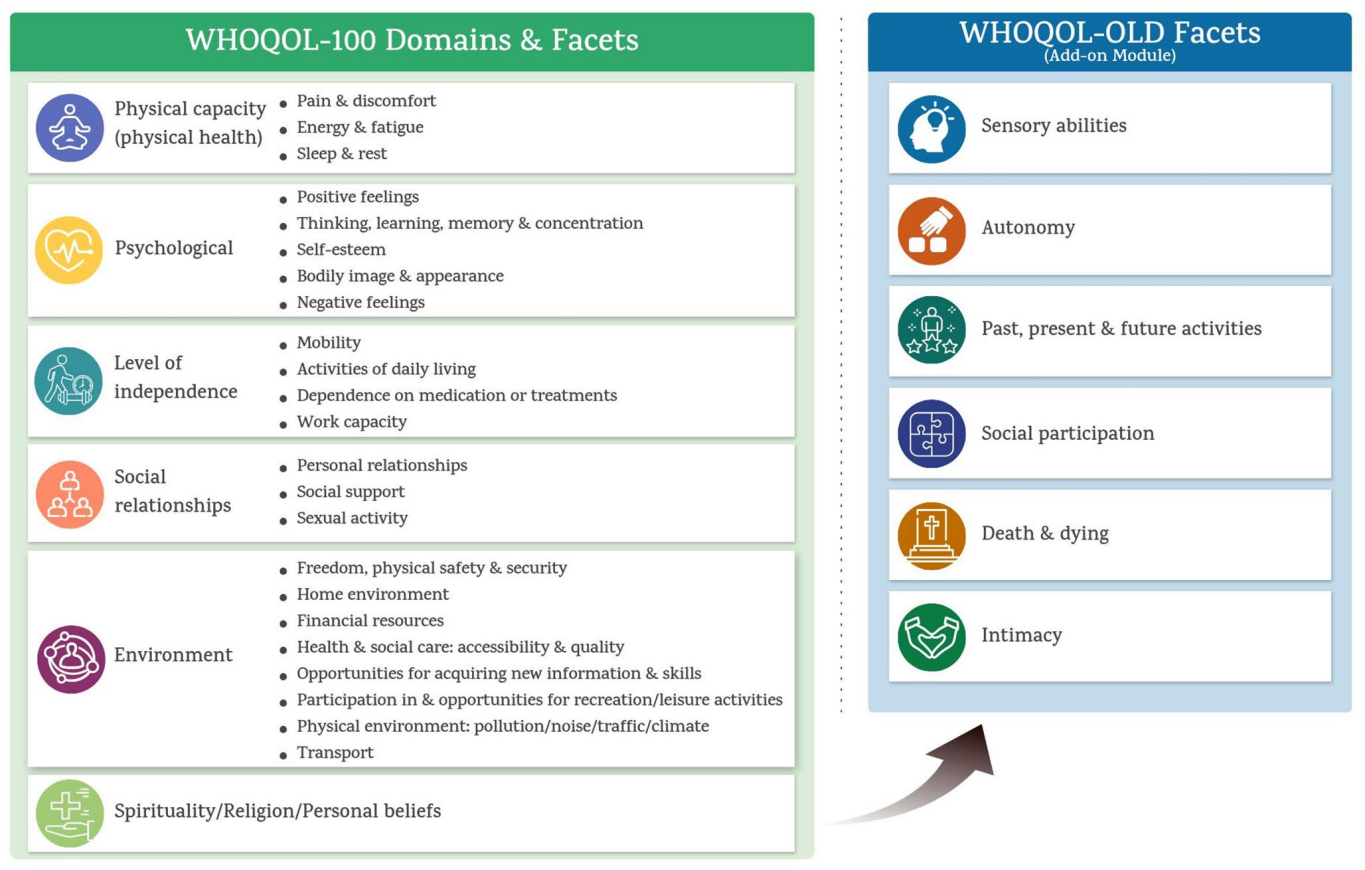
Domains and facets of the WHOQOL-100 and WHOQOL-OLD frameworks

We included studies on adults aged ≥ 65 with hearing loss that directly compared the effects of hearing aids combined with an additional structured intervention to hearing aids alone. Structured interventions were defined as behavioral, educational, psychosocial, or rehabilitative programs specifically designed to supplement hearing aid use and intended to improve quality of life or its related domains. The primary outcomes of interest were quality of life or its related domains, as conceptualized by the WHOQOL-100 and WHOQOL-OLD frameworks in Fig. 1 [6, 18], which provide a multidimensional approach suitable for evaluating quality of life in older adults with hearing loss. The WHOQOL-100 conceptualizes quality of life across six domains: physical capacity, psychological, level of independence, social relationships, environment, spirituality/religion/personal beliefs [6]. The WHOQOL-OLD framework is an age-specific add-on module to the WHOQOL framework, focuses on six age-relevant facets: sensory abilities, autonomy, past-present-future activities, social participation, death and dying, and intimacy [18]. These domains and facets were considered directly relevant to the present review, as hearing loss and hearing-related interventions may affect multiple aspects of later-life functioning, particularly sensory abilities, autonomy, psychological well-being, and social participation.

Studies were excluded if they:

1. did not include hearing aid users as the population of interest,
2. lacked a control group using hearing aids alone,
3. assessed outcomes not conceptually aligned with the WHOQOL-100 or WHOQOL-OLD domains,
4. included participants younger than 65 years without subgroup analysis, or
5. were feasibility or pilot studies not designed to evaluate intervention effectiveness.

Only randomized controlled trials (RCTs) were included, as we aimed to focus on the highest-quality evidence available to assess the effectiveness of interventions. Studies published in English from 1996 to 2024 were included, as 1996 marked the first commercial availability of digital hearing aids [19, 20]. A summary of the eligibility criteria is shown in Table 1.

**Table 1:** Study eligibility criteria

	Inclusion Criteria	Exclusion Criteria
Population	Older adults with hearing loss Participants over 65 years or with a mean age above 65 New and Experienced HA users	Participants under 65 years old Participants with other otologic disorders
Intervention	HAs with additional interventions	Studies involving forms of amplification other than HAs
Comparison	HAs alone	Studies focusing on medication or surgical interventions and HA-fitting methods
Outcome	Outcomes measured using quality of life instruments, along with outcomes that could be considered related to quality of life based on the domains of the WHOQOL-100 and WHOQOL-OLD	
Study design	Only RCT Non-qualitative studies	
Form of publication	Studies conducted between 1996 and 2024 Studies written in English	Conference abstract

HA; hearing aid, RCT; randomized controlled trial

### Search and study selection

An extensive literature search was performed through seven databases, including the Cochrane Library, CINAHL, PubMed, Embase, MEDLINE, Web of Science, and PsycINFO, which use terms related to older adults, hearing loss, hearing aids, interventions, and randomized controlled trials, as outlined in Table 2. Database-specific thesauri were applied, and the term intervention was included to capture a broad range of approaches. The search strategy was refined over two weeks with input from a medical librarian and finalized on October 6, 2024. Additional searches were conducted in the International Clinical Trials Registry Platform (ICTRP), ClinicalTrials.gov, PROSPERO, and grey literature through the Web of Science and PsycINFO. Three review authors independently screened the titles and abstracts via Rayyan, a widely used screening tool [21]. Full-text articles were reviewed for eligibility, with disagreements resolved by discussion. Inter-rater agreement among the three reviewers was substantial (Fleiss’ κ = 0.681). The rationale for study exclusion was documented at the full-text level and summarized in Supplementary Material S1.

**Table 2:** Outline of the search terms

Population		Intervention	Comparison	Study design
A	B	C	D	E
older adults aged elderly senior* pension* retire* geriatric*	hearing loss hearing impairment hard of hearing hearing defect auditory defect	hearing aid auditory appliance auditory prosthesis hearing device hearing apparatus listening aid	intervention program educat* train* rehabilitat* support communicat* counsel* improv* enhanc*	randomized controlled trial

The keywords within each category A through E were grouped using OR, and categories A through E were combined using AND. * Asterisks indicate search term truncation

### Data extraction

Three review authors manually extracted data using a standardized data extraction form. All data items were specified and agreed upon before data extraction to ensure consistency across reviewers. Disagreements were resolved through discussion, and study authors were contacted when clarification was necessary. The extracted data included study characteristics (publication year, authors, country, study aims, methods, and trial design), participant characteristics (age, gender, and duration of hearing aid use), intervention characteristics (type and content), follow-up period, outcome measures, quality-of-life-related outcomes, key findings, and funding sources.

### Outcomes and prioritization

This review examines how hearing aids combined with additional interventions improve the quality of life of older adults with hearing loss. We focused on outcomes related to quality of life on the basis of the WHOQOL-100 and WHOQOL-OLD domains, as shown in Table 3.

**Table 3:** Outcome domains and facets of quality of life (Power et al., 2005; WHO, 1998)

Domain	Facet	Description
Physical domain	Energy and fatigue	Assesses a person's energy and stamina for activities, ranging from experiencing fatigue to having adequate energy levels
Psychological domain	Positive feelings	Considers an individual's perspective of positive emotions as an essential component
	Negative feelings	Addresses the extent to which individuals experience negative emotions, including depression, anxiety, and a reduced sense of pleasure in life
	Self-esteem	Examines how individuals view themselves, spanning from positive to negative feelings, including aspects like self-efficacy, self-satisfaction, and control
Older-adult-specific domain	Sensory abilities	Identifies the impact of sensory impairments on daily life, participation in activities, and the ability to interact with others
	Autonomy	Evaluates whether individuals can make decisions, feel a sense of control, do what they want, and whether their freedom is respected by those around them

### Quality appraisal

Study quality was assessed using the Revised Cochrane Risk of Bias Tool (RoB 2). Three authors independently evaluated risk of bias using the Excel-based RoB 2.0 tool provided by the Cochrane risk-of-bias initiative, with disagreements resolved by consensus. Outcome reporting bias was evaluated by comparing study protocols with published results. Studies were categorized as low risk, some concerns, or high risk of bias based on algorithm-guided judgments from signalling questions rather than numerical scores. A study was classified as low risk of bias when all domains were judged as low risk. It was some concerns when at least one domain raised some concerns but no domain was judged as high risk, and high risk of bias when at least one domain was judged as high risk or when multiple domains with some concerns substantially lowered confidence in the results [22]. The certainty of evidence for the included studies was assessed using the Grading of Recommendations, Assessment, Development, and Evaluation (GRADE) framework. Evidence from randomized controlled trials was initially rated as high and downgraded, when appropriate, based on risk of bias, inconsistency, indirectness, imprecision, and publication bias, resulting in overall certainty ratings of high, moderate, low, or very low [23].

### Data synthesis

A wide variety of tools have been used to measure quality-of-life related outcomes. We classified and integrated these outcomes on the basis of the WHOQOL-100 and WHOQOL-OLD domains and facets. The reviewers carefully examined the individual items of each tool and classified them according to the domain of quality of life to which they were most closely aligned, based on conceptual relevance. Items were assigned to the domain that best represented their primary construct. Facets that primarily assessed functional status rather than quality-of-life aspects, or that were not explicitly measured by the instruments, were excluded from the final classification. Detailed domain and facet classifications are provided in Supplementary Material S2. Owing to the variety of measurement tools and statistical methods used, we reached out to ten authors for raw data, but only two responded. Due to the substantial heterogeneity among studies, primarily arising from differences in intervention content, duration, and outcome measures, a qualitative narrative synthesis was conducted [24]. Interventions were inductively categorized after study inclusion to facilitate synthesis. Two reviewers independently coded intervention characteristics using thematic analysis, with discrepancies resolved through consensus with a third reviewer. Results are summarized by intervention category in Table 4.

**Table 4:** Summary of included studies

No	Year	Author	Country	Sample size	Experimental Group	Control Group	Intervention defined
1	2002	Abrams, H., et al	USA	105	53 Age in years (Mean ± SD): 74.5 ± 6.9 Gender (n, M/F): 31/22 PTA (dB HL, Mean ± SD): 35.1 ± 11.8 (right ear) and 35.7 ± 11.5 (left ear)	52 Age in years (Mean ± SD): 73.0 ± 7.6 Gender (n, M/F): 36/16 PTA (dB HL, Mean ± SD): 34.5 ± 12.3 (right ear) and 32.1 ± 12.0 (left year)	Group-Based Auditory Rehabilitation
2	1997	Andersson, G., et al	Sweden	19	9 Age in years (Mean ± SD): N/A Gender (n, M/F): 5/4	10 Age in years (Mean ± SD): N/A Gender (n, M/F): 6/4	Individual at-Home Training
3	2017	Armitage, C. J., et al	UK (England)	50	25 Age in years (Mean ± SD): 71.60 ± 12.15 Gender (n, M/F): N/A	25 Age in years (Mean ± SD): 67.44 ± 13.51 Gender (n, M/F): N/A	Empowerment Strategy
4	1994	Cherry, R. and A. Rubinstein	USA	33	17 Age in years (Mean ± SD): 76.6 ± 8.1 Gender (n, M/F): N/A PTA (dB HL, Mean ± SD): 43.0 ± 14.8 HA use (n, New/Previous ): 17/13	16 Age in years (Mean ± SD): 74.1 ± 8.5 Gender (n, M/F): N/A PTA (dB HL, Mean ± SD): 38.0 ± 13.3 HA use (n, New/Previous ): 16/14	Telehealth
5	2024	Han, J. S., et al	Korea	39	19 Age in years(Mean ± SD): 70.5 ± 6.9 Gender (n, M/F): 8 / 12 HL duration in months (Mean ± SD): 107.9 ± 83.3 HA use duration in months (Mean ± SD): 56.1 ± 72.2	20 Age in years(Mean ± SD): 69.5 ± 13.8 Gender (n, M/F): 8 / 13 HL duration in months (Mean ± SD): 98.3 ± 91.3 HA use duration in months (Mean ± SD): 53.0 ± 50.7	Individual at-Home Training
6	2019	Humes et al	USA	43	13 Age in years(Mean ± SD): 71.9 ± 6.1 Gender (n, M/F): 9/4 PTA (dB HL, Mean ± SD): 36.0 ± 12.3 (right ear) 34.7 ± 12.1 (left ear)	Active Control (AC) = 15 Passive Control (PC) = 15 < AC > Age in years(Mean ± SD): 71.3 ± 7.5 Gender (n, M/F): 11/4 PTA (dB HL, Mean ± SD): 29.1 ± 15.2 (right ear) 31.1 ± 15.8 (left ear) < PC > Age in years(Mean ± SD): 72.0 ± 7.1 Gender (n, M/F): 11/4 PTA (dB HL, Mean ± SD): 28.0 ± 16.8 (right ear) 31.1 ± 16.0 (left ear)	Individual at-Home Training
7	2005	Kramer, S. E., et al	Netherland	48	24(Hearing Impaired, HI) + 24(Significant Others) < HI > Age in years(Mean ± SD): 69 ± 7.7 Gender (n, M/F): 16/8 PTA (dB HL, Mean ± SD): 53.7 ± 13.3 HA use (n, first/experienced): 12/12	24(Hearing Impaired, HI) + 22(Significant Others) < HI > Age in years(Mean ± SD): 71 ± 8.5 Gender (n, M/F): 12/12 PTA (dB HL, Mean ± SD): 56.3 ± 15.7 HA use (n, first/experienced): 9/15	Group-Based Auditory Rehabilitation
8	2024	Lelic, D., et al	Denmark	21	11 Age in years: 68 ± 7 Gender (n, M/F): 8/3 HA experience in years (Median [Range]): 13 [1-37]	10 Age in years: 64 ± 8 Gender (n, M/F): 7/3 HA experience in years (Median [Range]): 12.5 [1-53]	Empowerment Strategy
9	2020	Meijerink, J. F. J., et al	Netherland	343	180 Age in years (Mean ± SD): 68.1 ± 8.4 Gender (n, M/F): 108/72	163 Age in years (Mean ± SD): 68.2 ± 8.7 Gender (n, M/F): 98/65	Individual at-Home Training
10	2013	Olson, A. D., et al	USA	29	8 (New HA users + Training) Age in years (Mean ± SD): 66 ± 9.6 Gender (n, M/F): 4/4 HL duration: 10.2 (SD = 10.3) 14 (Experienced HA users + Training) Age in years (Mean ± SD): 68 ± 6.9 Gender (n, M/F): 4/10 HL duration: 16.4 (SD = 11.8)	7 (No training, New HA use only) Age in years (Mean ± SD): 66 ± 9.3 Gender (n, M/F): 6/1 HL duration: 11.4 (SD = 10.2)	Individual at-Home Training
11	2006	Stecker, G. C., et al	USA	31	Experiment 1 (New HA users) 12 Age in years (Mean [Range]): 69 [50–80] Gender (n, M/F): N/A Experiment 2 (Experienced HA users) 8 Age in years (Mean [Range]): 67.7 [61–75] Gender (n, M/F): N/A HA experience in months (Mean [Range]): 16 [10–21]	Experiment 1 (New HA users) 11 Age in years (Mean [Range]): N/A Gender (n, M/F): N/A Experiment 2 (Experienced HA users) None	Individual at-Home Training
12	2020	Vreeken, H. L., et al	Netherlands and Belgium	131	64(Participants) + 57(Communication Partners) < Participants > Age in years(Mean ± SD [Range]): 81.3 ± 9.9 [56–97] Gender (n, M/F): 37/27 DSL duration in years (Median [IQR]): 4.0 [2–7]	67(Participants) + 53(Communication Partners) < Participants > Age in years(Mean ± SD [Range]): 81.9 ± 10.0 [53–99] Gender (n, M/F): 26/41 DSL duration in years (Median [IQR]): 5.5 [2.75–10]	Group-Based Auditory Rehabilitation

No	Intervention description	Facet of Outcome	Outcome measure	Time points (time frame)	Major findings	Funding	Rob2 Consensus
1	A group-based audiologic rehabilitation program approach to improving communication for the hard of hearing, covering auditory processes, strategies for challenging listening environments, anticipatory and repair techniques, and resources like assistive technology and community support for group	Overall quality of life	SF-36 V	4 weeks (Immediate): Post-intervention assessment	Greater improvement in mental health (MCS score) (p = 0.01) compared to HA alone	N/A	High
2	Received coping skills training at home to enhance communication strategies and manage hearing-related difficulties	1. Autonomy 2. Sensory abilities	1. CPHI, HCA 2. VAS	1wk (Immediate)	Improved coping behaviors and reduced hearing problems in elderly individuals with minimal therapist involvement, better stress management and communication skills, but no significant changes in daily HA use	Swedish Council & Sasakawa Fund	High
3	Self-affirmation was induced at baseline using an established manipulation that was embedded at the end of the research questionnaires	1. Negative feelings 2. Self-esteem	1. Lasher and Faulkender’s anxiety about ageing scale 2. Ad hoc research tool	10 wks	Reduction in anxiety levels and improved acceptance of aging (p < 0.01), compared to the control group. HA use was higher in the intervention group but not statistically significant	Manchester NHS Trust	Some concerns
4	Periodic clinician-initiated telephone contact whether HA users were experiencing any problems (e.g., feedback, discomfort, handling of aid, etc.). Problems were addressed through trouble-shooting (e.g., dead batteries) and counseling over the phone, or if indicated, subjects were scheduled for a clinic appointment	1. Positive feelings 2. Negative feelings	1. Ad hoc research tool 2. Ad hoc research tool, HHIE	6wks (Immediate): Follow-up at 6wks post fitting 9wks (Intermediate): Follow-up at 9wks post fitting 12wks (Intermediate): Follow-up at 12wks post fitting 4mths (Intermediate): Final interview at 4mths	The intervention group had more frequent identification and resolution of hearing aid-related problems. But no statistically significant in terms of satisfaction or perceived benefit	CUNY Grant	High
5	Chat based mobile training contains dual auditory and word training program. Encouraged to use CMAT daily for 8 weeks (2 months). One CMAT session consisted of 20 questions, took about 5 to 10 min, and was repeated 3 times a day (30 min a day)	1. Positive feelings 2. Negative feelings 3. Sensory abilities	1. K-IOI-HA 2. APHAB, K-HHIE 3. Ling Six Sound test, VCIT, Monosyllable, and bi-syllable open-set tests, K-CID	1mth (Immediate) 2mths (Intermediate)	Significant improvements in word and sentence perception (p = 0.04, p = 0.03, respectively) compared to HA alone	Korea SME Ministry	High
6	Self-administered intervention at home, frequent-word auditory training. Received words, phrases, and sentences in backgrounds of noise and trial-to-trial feedback on their identification performance Instructions were given to complete 3 sessions per week, for a total of 15 at-home sessions	1. Positive feelings 2. Negative feelings 3. Sensory abilities	1. HASS 2. HHIE 3. CST, CID, Trained frequent-word speech materials, PHAP, ANL	5 ~ 6wks (Immediate) 10 ~ 11wks (Intermediate) 15 ~ 16wks (Intermediate) 21 ~ 22wks (Intermediate)	Participants who engaged in frequent auditory training showed greater improvements in word recognition(p < 0.05) in noise compared to HA alone	NIH NIDCD Grant	Low
7	Training in communication strategies with significant others, speechreading, information on how to use hearing aids, and details on additional technical devices A self-administered intervention comprised of 5 videotapes and an instruction booklet Each video depicts common daily challenges faced by the elderly, demonstrating communication strategies to improve interactions and raise awareness about hearing loss and its impact	1. Positive feelings 2. Negative feelings	1. IOI-HA, IOI-AI 2. Items derived from HHDI	Immediately after the program (Immediate) 6 mths (Intermediate)	Increased awareness of the benefits of speechreading and improved interaction with their significant others (p < 0.05). The control group experienced a decrease in quality of life, whereas the intervention group showed improvements at the 6-month follow-up. No significant group differences were observed regarding emotional response(p > 0.05)	ZonMw & Sluyterman Foundation	High
8	Asked to report their positive listening experiences via an app (for 3 weeks)	1. Positive feelings 2. Sensory abilities 3. Autonomy	1. Ad hoc research tool, IOI-HA, HEARLI-Q, EMA 2. COSI for "final ability" 3. COSI for "degree of change"	1wk (Immediate) 4wks (Immediate)	Increased satisfaction and perceived benefit from hearing aids (COSI)(p < 0.05) compared to HA alone	N/A	High
9	A practical support booklet and online materials were delivered via email throughout their 6-month HA rehabilitation trajectory The online materials included 3 instruction videos on HA handling, 5 videos on communication strategies, and 3 testimonial videos	1. Autonomy 2. Positive feelings 3. Self-esteem 4. Negative feelings	1. CPHI 2. IOI-HA, IOI-AI 3. MARS-HA, URICA-HL 4. AIADH	6mths (Intermediate) 12mths Intermediate)	Significantly greater self-efficacy for advanced HA handling (p = 0.04) and hearing aid satisfaction(p = 0.006) compared to HA alone	AudioNova & VU Medical Center	Some Concerns
10	Auditory training (AT) at home using the LACE DVD 20 lessons from the LACE DVD program over 4 weeks	1. Sensory abilities 2. Positive feelings	1. QuickSIN, Compressed Speech Test, SSI, SSQ 2. IOI-HA, IOI-AI	2wks (Intermediate): Midpoint assessment after 2wks of training 4wks (Intermediate): Post-training assessment	Improvements in speech recognition(p < 0.05) compared to HA alone	University of Kentucky	High
11	8 weeks of at-home syllable identification training	Sensory abilities	NST	1wk (Baseline): Initial assessment 2wks (Intermediate): Mid-training assessment 4wks (Intermediate): Post-training assessment 16wks (Long-term): Retention test after training	Significant improvement in syllable identification and phoneme recognition(p < 0.001) compared to HA alone	VA Research Grant	High
12	Participants and their communication partners received occupational therapy ‘Dual Sensory Loss Protocol’ for effective communication strategies as a group The protocol consisted of: (1) optimal use of HAs; (2) use of assistive devices and adaptations to the living environment; and (3) coping with DSL and use of effective communication strategies	1. Energy and fatigue 2. Negative feelings 3. Autonomy	1. FAS 2. CES-D, De Jong Gierveld Loneliness Scale 3. CPHI	3mths (Intermediate)	Significant effect (effect size SMD = 0.60, 95% CI: 0.25–0.95) in the use of verbal communication strategies (CPHI), but not significant after adjusting confounders. No significant overall improvements in HA use	ZonMw, Oogfonds, Capita	Some Concerns

AIADH, Amsterdam Inventory for Auditory Disability and Handicap; ANL, Acceptable Noise Level; APHAB, Abbreviated Profile of Hearing Aid Benefit; CES-D, Center for Epidemiological Studies – Depression Scale; CID, Central Institute for the Deaf sentence materials; CMAT, Chat-based Mobile Auditory Training; COSI, Client-Oriented Scale of Improvement; CPHI, Communication Profile for the Hearing Impaired; CST, Connected Speech Test; dB HL, decibels hearing level; DSL, Dual Sensory Loss; EMA, Ecological Momentary Assessment; FAS, Fatigue Assessment Scale; HA, Hearing Aid; HASS, Hearing Aid Satisfaction Survey; HCA, Hearing Coping Assessment; HEARLI-Q, Hearing-Related Lifestyle Questionnaire; HHDI, Hearing Handicap and Disability Inventory; HHIE, Hearing Handicap Inventory for the Elderly; HL, Hearing Loss; IOI-AI, International Outcome Inventory for Alternative Interventions; IOI-HA, International Outcome Inventory for Hearing Aids; IQR, Interquartile Range; K-CID, Korean version of Central Institute for the Deaf test; K-HHIE, Korean version of the Hearing Handicap Inventory for the Elderly; K-IOI-HA, Korean versions of the International Outcome Inventory for Hearing Aids; LACE, Listening And Communication Enhancement; MARS-HA, Measure of Audiologic Rehabilitation Self-Efficacy for Hearing Aids; NST, Nonsense Syllable Test; OT, Occupational Therapists; PHAP, Perception of Hearing Aid Performance; PTA, Pure Tone Average calculated for 'the better ear' over the frequencies of 0.5 ~ 4 kHz; QuickSIN, Quick Speech in Noise; SD, Standard Deviation; SF-36 V, 36-Item Short-Form Health Survey modified for the Veteran population; SSI, Synthetic Sentence Identification; SSQ, Speech, Spatial and Qualities of Hearing Scale; URICA-HL, University of Rhode Island Change Assessment adapted for Hearing Loss; VAS, Visual Analogue Scales; VCIT, Vowel and Consonant Imitation Test

## Results

### Study selection

**Fig. 2:**
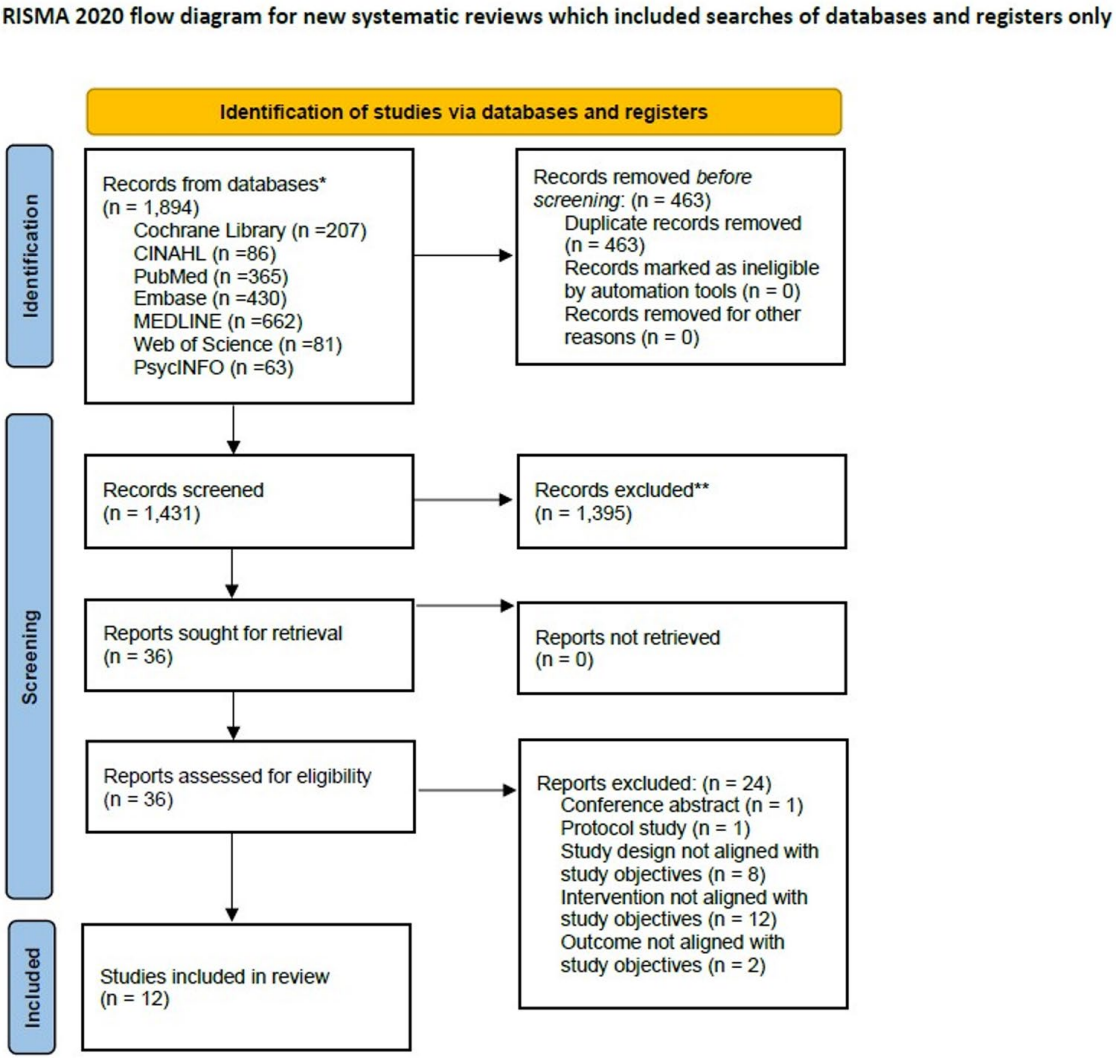
PRISMA 2020 flow diagram

We found a total of 1,894 records from seven databases, and after removing 463 duplicates, 1,431 records remained. After screening the titles and abstracts, 1,395 articles were excluded. Subsequently, 36 studies were fully screened by reviewing the full texts. Of these, 24 articles were excluded because they were nonrandomized controlled trials, were study protocol articles, lacked hearing aid users in the control group, intervention not aligned with study objectives, or reported outcomes not aligned with study objectives. Finally, 12 studies that met the study inclusion criteria were selected for this review. Our research selection process is summarized in Fig. 2.

### Quality assessment

**Fig. 3:**
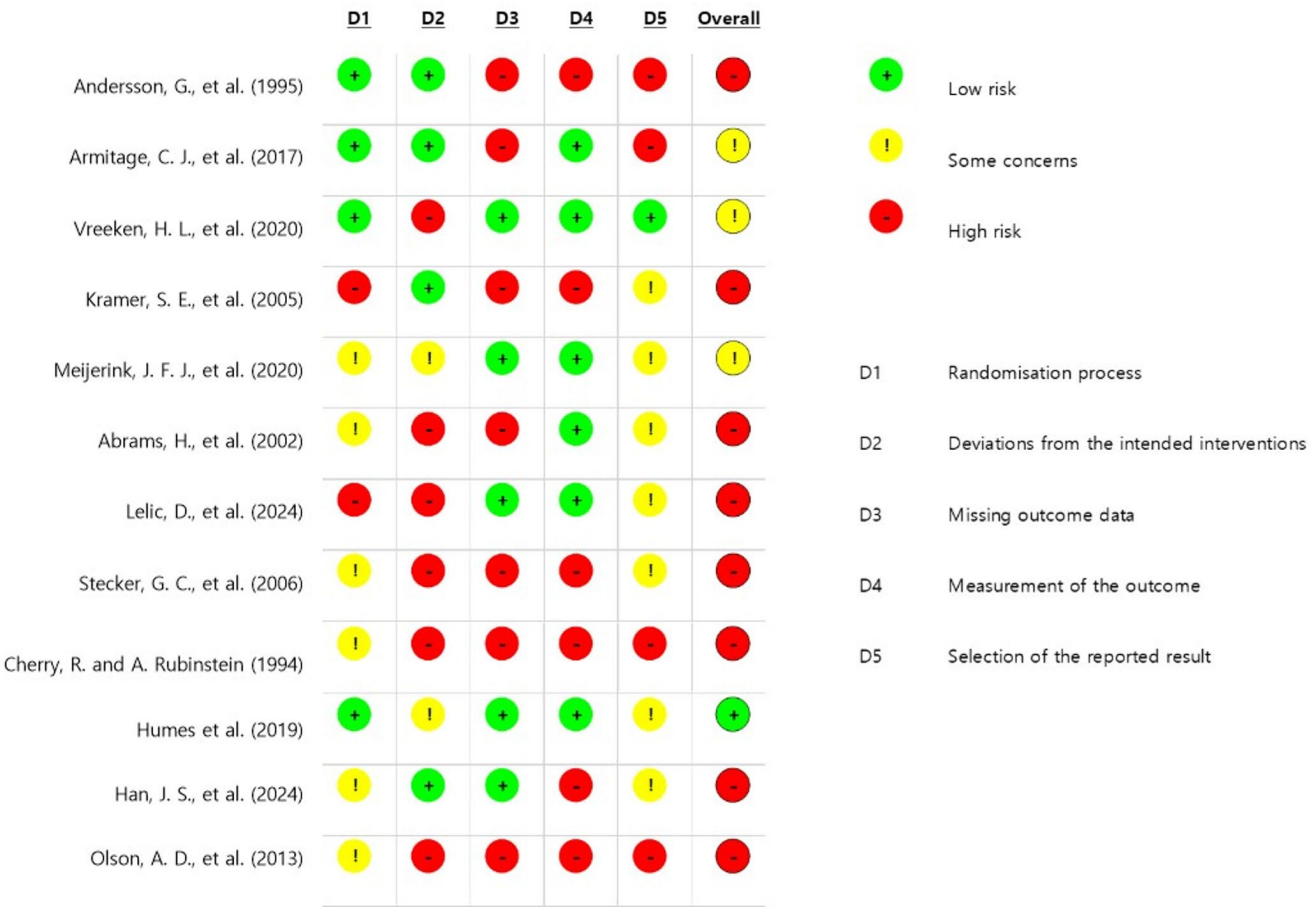
Quality assessment results for individual studies for each and overall domain

The results of the evaluation are shown in Fig. 3. The quality of the studies was graded as “high risk” across the other domains, except for the randomization process domain. After the results from all five RoB 2 domains (bias arising from the randomization process, deviations from intended interventions, missing outcome data, measurement of the outcome, and selection of the reported result) were evaluated, eight studies were classified as “high risk" in quality, three as "some concerns," and one as "low risk." No studies were excluded on the basis of these quality assessments to ensure the comprehensive inclusion of all available evidence, thereby strengthening the overall synthesis.

### Study characteristics

The published dates of the studies ranged from 1994 to 2024 in 7 countries, with most of the studies from Europe (n = 6); the Netherlands (n = 2), Denmark (n = 1), Belgium (n = 1), Sweden (n = 1), and the United Kingdom (n = 1). Additionally, five studies were from North America (United States, n = 5), and one study was from Asia (Korea, n = 1). All studies were randomized controlled trials. The sample sizes in all studies varied from 19 to 343 study participants, with a total of 892 participants across all included studies. The participants were older adults, with the experimental group aged between 64 and 81.3 years and the control group aged between 66 and 81.9 years. Both participants predominantly had bilateral sensorineural hearing loss ranging from mild-to-moderate across the studies. The severity of hearing impairment is typically measured by pure-tone audiometry, with better-ear average thresholds ranging from 30 to 60 dB HL. Additionally, other baseline testing measures, such as speech-in-noise capabilities [25–27], the National Hearing Test (NHT), the Connected Speech Test (CST), and the Nonsense Syllable Test (NST), have also been utilized in several studies [11, 26, 27]. Table 4 presents the detailed information.

All participants in the control group were hearing aid users. Several studies ensured the balanced inclusion of both first-time hearing aid users and those who have already used hearing aids. These studies evenly distributed participants across interventions involving both new and long-term users [25, 26, 28, 29]. Many studies focused on participants who have already used hearing aids [11–13, 27, 30]. In contrast, some research has specifically targeted new hearing aid users to address their acclimatization challenges [14, 31, 32].

Three studies divided participants into three groups [25–27]: (1) one experimental group and two control groups (passive and active) [26]; (2) one control group consisting of new hearing aid users who did not receive the additional intervention, and two intervention groups consisting of new hearing aid users who received the intervention and experienced hearing aid users who received the intervention [25]; and (3) experimental and control groups of new hearing aid users, and an experimental group of experienced hearing aid users [27]. Additionally, two studies included the partners of participants to evaluate their interactions with the study participants and the efficacy of the intervention [11, 29].

### Interventions

The interventions were thematically clustered into four categories through iterative consensus discussions to reflect common conceptual objectives: (1) individual at-home training (n = 6), which had the highest proportion, followed by (2) group-based auditory rehabilitation (n = 3), (3) empowerment strategies (n = 2), and (4) telehealth (n = 1) in that order. These categories were determined after study inclusion to help synthesize similar approaches.

First, individual at-home training included coping skills and speech recognition training at home. The training reduced maladaptive behaviors and hearing-related problems [28], and improved speech-in-noise comprehension and communication, with greater benefits observed among new hearing aid users [25]. Chat-based auditory training improved word/sentence perception but not phoneme/consonant perception [12]. Participants reported significantly higher NST scores on laboratory-based phoneme identification tasks in noise compared with hearing aids alone [27], with gains maintained for up to 8.5 months but limited to trained materials [26]. Although emotional responses and communication strategies improved, between-group differences were not significant. However, self-efficacy in hearing aid handling and hearing aid satisfaction improved, as measured by Measure of Audiologic Rehabilitation Self-Efficacy for Hearing Aids (MARS-HA) and the International Outcome Inventory for Hearing Aids (IOI-HA), respectively. [13].

Second, group-based auditory rehabilitation included posthearing aid fitting programs (e.g., hearing aid use, communication strategies) and comprehensive communication training (e.g., raising problem awareness about hearing loss and enhancing communication). The training group showed enhanced communication strategies, quality of life and satisfaction following the treatment, whereas no such improvement was noted in the control group. The training group, unlike the control group, sustained enjoyment of life [29]. However, some studies reported no significant differences between the intervention group and the control group [11, 31].

Third, the empowerment strategy included self-affirmation (e.g., writing resolutions such as “If I feel anxious, I’ll remember past successes”) and reporting positive listening experiences. Self-affirmation reduces aging-related anxiety and improves aging acceptance but does not significantly affect self-efficacy [14]. Reporting positive listening experiences significantly improved satisfaction, perceived benefits, listening ability, and the degree of change, with a positive correlation observed between coping ability and positive reporting [30].

Finally, telehealth provided remote support through telephone (e.g., addressing problems with hearing aids). Telephone consultations did not reduce unresolved issues or increase patient satisfaction [32].

### Outcomes

**Fig. 4:**
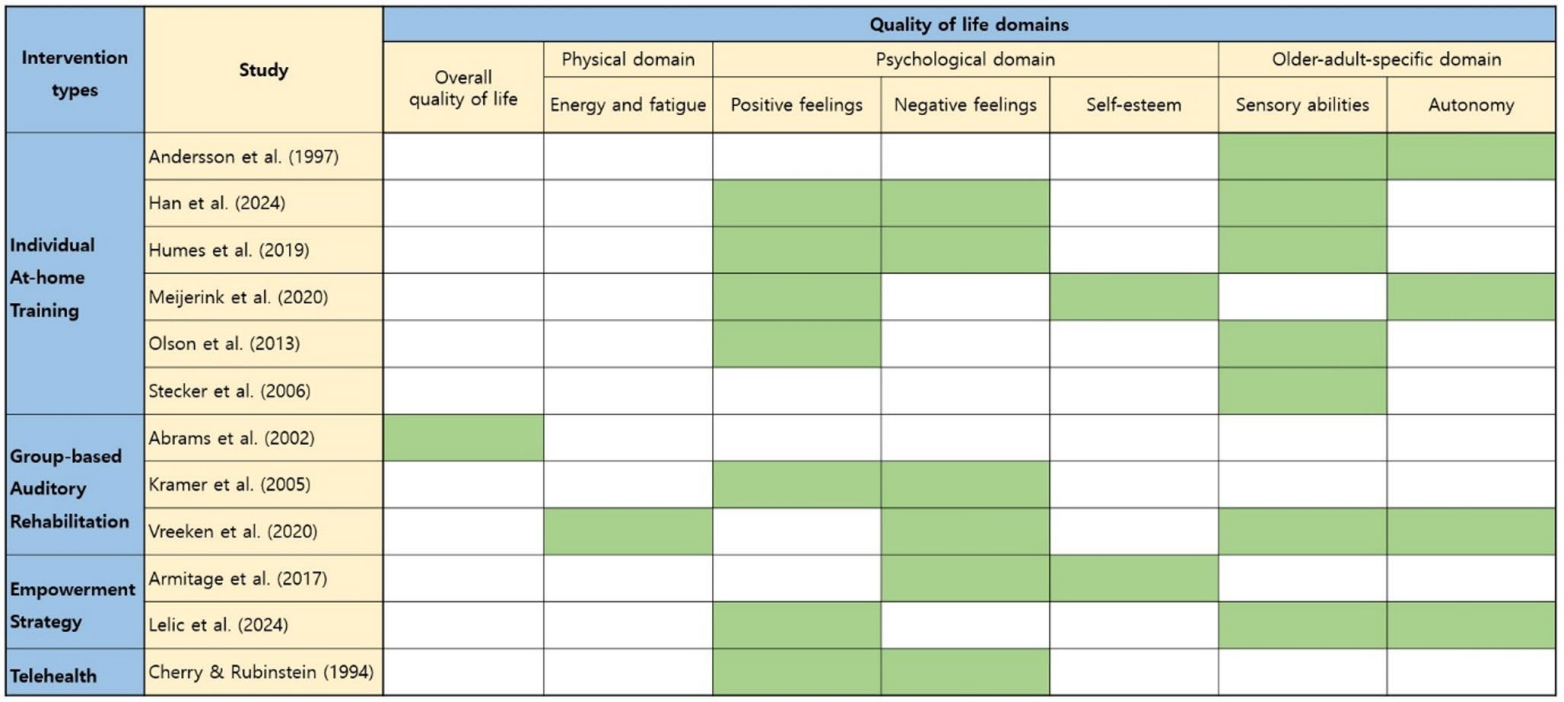
Quality of life domains identified by types of interventions in the studies

Among the 12 included studies, only one study explicitly used a quality of life measure (i.e., the 36-Item Short-Form Health Survey modified for the Veteran population (SF-36 V)) to evaluate how additional auditory interventions impact older hearing aid users’ overall quality of life [31]. Three quality-of-life domains[6, 18], including six facets, were identified on the basis of the WHOQOL-100 and WHOQOL-OLD domains. Nine studies (75%) reported outcomes in the psychological domain [11–14, 25, 26, 29, 30, 32], with six (50%) addressing multiple facets of the psychological domain [12–14, 26, 29, 32]. Among them, four evaluate positive and negative feelings [12, 26, 29, 32]. Eight studies (67%) focused on the older adult-specific domain [11–13, 25–28, 30], with three (50%) covering both sensory ability and autonomy [11, 28, 30]. The physical domain (e.g., energy and fatigue) was assessed in one study [11], which was also the only study covering all physical, psychological, and older-adult-specific domains. Figure 4 illustrates the quality of life domains identified according to the types of interventions and individual studies.

### Measurement tools

The included studies utilized a diverse range of outcome measures to assess the effectiveness of interventions, particularly in terms of quality of life.

In the psychological domain of quality of life, the IOI-HA was the most frequently used measure and was used in four studies [13, 25, 29, 30] to assess positive feelings such as satisfaction and perceived benefits. The Hearing Handicap Inventory for the Elderly (HHIE), used in three studies [12, 26, 32], was employed to assess negative feelings, particularly perceived hearing handicap. Self-esteem was measured via tools such as the MARS-HA and the University of Rhode Island Change Assessment adapted for Hearing Loss (URICA-HL), both of which are applied in one study [13].

In the older-adult-specific domain of quality of life, the Communication Profile for the Hearing Impaired (CPHI), which appeared in three studies [11, 13, 28], evaluated autonomy by examining communication strategies and personal adjustment to hearing loss. Sensory abilities were assessed via various tools. For example, speech recognition in noise was assessed through the Quick Speech In Noise (QuickSIN) test [25], the CST [26], and the Client-Oriented Scale of Improvement (COSI), which assesses improvements in auditory processing and communication abilities [30]. Additionally, syllable identification training and listening enhancement programs incorporated tests such as the NST [27].

Notably, only one study utilized the Fatigue Assessment Scale (FAS) to measure energy and fatigue in physical domain of quality of life [11].

### Certainty of evidence

The certainty of evidence for each outcome was assessed using the GRADE approach. Outcomes reported by only one randomized controlled trial (e.g., overall quality of life and physical domain), the certainty of evidence was downgraded for serious imprecision and inability to assess inconsistency. Evidence from specific populations (e.g., veterans) was downgraded for indirectness due to limited generalizability, and small sample sizes contributed to further downgrading. Publication bias was not formally assessed because of the small number of studies and was considered within the overall judgments. As summarized in Supplementary Material S3, certainty of evidence was rated as very low for overall quality of life and low for the physical, psychological, and older-adult-specific domains, indicating limited confidence in the estimated effects and the need for cautious interpretation.

## Discussion

This review synthesized the characteristics, interventions, outcomes, and major findings of studies investigating additional interventions used with hearing aids. Across 12 randomized controlled trials published between 1996 and 2024, four intervention categories were identified: individual at-home training, group-based auditory rehabilitation, empowerment strategies, and telehealth. These findings highlight the potential benefits and limitations of supplementing hearing aid use and identify factors that may support adaptation, participation, and emotional well-being in everyday listening contexts.

### Individual at-home training

Individual at-home training, the most prevalent type of intervention (six studies), showed partial improvements in positive feelings, self-esteem, sensory abilities, and autonomy [12, 13, 25–28]. These findings suggest that such training with hearing aids may increase satisfaction, self-efficacy, partial auditory abilities, and coping skills, albeit with low certainty. This aligns with prior research indicating that individual training fosters greater learning and skill development [33]. However, discomfort and negative emotions related to hearing loss generally persisted, indicating that individual training alone may be insufficient to address the emotional and interpersonal challenges of hearing impairment. Limited opportunities for real-world communication practice and interaction, which are typical of group-based rehabilitation, may explain the modest improvements in communication with others.

Hearing loss is associated with emotional distress and reduced emotional well-being [34], often manifesting as frustration, grief, and burdensomeness while communicating with others [35]. Emotional well-being also depends on accurate perception of one’s own and others’ emotions, which may be impaired by hearing loss, further exacerbating its impact [36]. Nevertheless, evidence that sensory gains were maintained for over eight months for trained materials suggests that targeted training using difficult words or sentences may produce sustained improvements in communication within trained contexts. Future longitudinal studies are needed to determine whether such benefits generalize to untrained language content.

### Group-based auditory rehabilitation

Group-based auditory rehabilitation has positive impacts on both psychological and older adult-specific domains, including increased autonomy and some improvement in positive feelings [11, 29, 31]. These benefits may stem from peer support and shared experiences, which can reduce isolation and foster mutual understanding [37]. Previous studies have shown that participation in group auditory programs can reduce self-perceived hearing difficulties and improve communication skills and hearing aid use [38, 39]. The opportunity to learn and practice communication strategies in a supportive, real-world context may underlie these effects.

However, no consistent improvements were observed in overall quality of life, energy and fatigue, or negative feelings in this review. Mixed findings and potential challenges related to group dynamics, such as limited trust among participants, may have constrained effectiveness [40]. These variations highlight the need for further research to identify the most effective components of group-based interventions.

### Empowerment strategy

Empowerment strategies, such as self-affirmation and sharing positive listening experiences, showed promising effects, particularly in the psychological domain [14, 30]. These strategies, which are linked to positive thinking and inner strength [41], may foster psychological empowerment by reducing anxiety and negative emotions related to aging with hearing loss and promoting positive adaptation. Evidence suggests that self-affirmation can increase receptivity to health-related information, supporting subjective well-being in the context of hearing loss [42].

Additionally, empowerment strategies may enhance self-reliance and inner resilience [43], contributing to the autonomy aspect of the older adult-specific domain. Such psychological resilience has been found to play a significant role in mediating the association between hearing impairment and social well-being in older adults. A study showed that psychological resilience accounted for 50.9% of the variance in the change in social well-being for individuals with hearing loss [44]. However, the lack of significant effects on self-efficacy in this study indicates the need for further research to refine these strategies, particularly in addressing self-esteem and other psychological aspects of quality of life.

### Telehealth

Telehealth interventions, specifically telephone support, showed limited evidence of improving quality of life in older adults with hearing loss and were associated with low certainty. Previous studies reported no significant reductions in unresolved issues or improvements in satisfaction with telephone-only support, suggesting that it may not adequately address complex needs, whereas video-based telehealth showed more favorable cognitive and mental health outcomes [32, 45].

However, telehealth remains a promising avenue for healthcare delivery, particularly given its feasibility and ability to reach underserved populations. Studies have shown that telehealth can reduce barriers to access, lower medical costs, and improve chronic disease management among older adults [46, 47]. Digital health technology also enables more person-centered care beyond visit-based models, with remote hearing aid fitting showing satisfaction comparable to in-person services and artificial intelligence-based systems explored for self-fitting of hearing aids [48]. Given the growing reliance on remote healthcare solutions, further research is needed to explore modern telehealth applications, such as video consultations or hybrid models, in addressing the specific needs of older adults with hearing loss. Challenges related to digital literacy, cognitive ability, and user experience highlight the need for tailored support and improved provider communication skills [49]. Future studies should focus on enhancing accessibility, individualizing interventions, and evaluating the long-term effects of telehealth on quality of life.

### Overall impact on quality-of-life domains

Reviewers examined each item and assigned it to the quality-of-life domain that best reflected its primary construct based on conceptual relevance. Items assessing functional status rather than quality of life, or not explicitly measured, were excluded from the final classification. The analysis revealed a strong focus on the psychological and older adult-specific domains, with less attention given to the physical aspects of quality of life. A significant proportion of studies (58.3%) focused on sensory abilities [11–13, 25, 27, 28, 30], positive feelings (58.3%) [12, 13, 25, 26, 29, 30, 32], and negative feelings (58.3%) [11–14, 26, 29, 32], highlighting their importance for older adults with hearing loss. However, autonomy (33.3%) [11, 13, 28, 30] and self-esteem (16.7%) [13, 14] were less emphasized, suggesting that these aspects may be underexplored in auditory rehabilitation research.

Furthermore, minimal attention to energy and fatigue (8.3%) [11] and overall quality of life (8.3%) [31] indicates that broader physical and global quality-of-life outcomes are largely neglected in the current evidence base. Although auditory and psychological outcomes appear to be prioritized in existing interventions, these findings suggest that a more comprehensive and balanced assessment across WHOQOL domains may be necessary to better capture the full impact of interventions on the lives of older adults with hearing loss.

### Measurement tools

The diverse range of measurement tools used across the studies highlights the multifaceted nature of the interventions and the outcomes they aim to assess, particularly in relation to quality of life. The frequent use of tools such as the IOI-HA and the HHIE suggest that assessing both positive and negative emotional responses, particularly satisfaction and perceived hearing handicap, is central to understanding the effectiveness of interventions in improving quality of life [29, 30, 32]. In line with research by Humes et al. [26], emotional responses to hearing interventions, such as satisfaction and perceived benefits, directly influence quality-of-life outcomes by improving social participation and reducing feelings of isolation and frustration. These tools are valuable in evaluating how interventions impact individuals' psychological well-being and their perceived quality of life.

The use of measures such as the CPHI and self-esteem scales (MARS-HA, URICA-HL) underscores the importance of assessing autonomy and personal adjustment to hearing loss [13, 28]. These findings highlight the role of autonomy and self-efficacy in quality of life among individuals with hearing loss. Recent clinical recommendations in audiologic rehabilitation emphasize that addressing social-emotional well-being and individualized goals, including patients’ sense of control and adaptive capacity, is essential for supporting adjustment and overall well-being [50]. Accordingly, effective interventions should address not only sensory function but also personal agency to reduce the psychological burden of hearing loss.

The variety of sensory ability assessments, such as the QuickSIN, CST, and COSI, reflects the complex nature of auditory processing and communication challenges faced by individuals with hearing loss [25, 26, 30]. These tools are crucial in capturing improvements in auditory capabilities, which have been shown to positively influence quality of life by enhancing communication in social, educational, and professional settings. Moreover, objective measures of auditory function combined with subjective measures, as suggested by Wang et al. [51], are essential for understanding the tangible benefits of hearing interventions and their acceptance and satisfaction, which are directly linked to overall life satisfaction.

Interestingly, the use of the FAS in only one study [11] implies that energy and fatigue may be underexplored aspects of the impact of hearing loss on quality of life. Fatigue, often a consequence of hearing loss and the effort required for communication, can have a profound effect on overall well-being and quality of life. This finding suggests that future research should incorporate fatigue measures into quality-of-life assessments. One study emphasized the importance of understanding how hearing loss-related fatigue affects emotional and physical well-being and the ability to engage in daily activities [52]. The incorporation of fatigue into future studies could provide a more comprehensive understanding of how hearing interventions affect overall life satisfaction and holistic quality of life.

Overall, the variety of tools used across these studies highlights the need for a multidimensional approach for evaluating the effectiveness of hearing loss interventions in terms of quality of life. Future research should continue to integrate both objective and subjective measures to capture the full impact of interventions to enhance our understanding of the comprehensive benefits these interventions provide to individuals' overall well-being.

### Limitations and strengths

Several limitations should be considered when interpreting the findings of this review. First, the diversity of interventions complicates direct comparisons. The WHOQOL-100 and WHOQOL-OLD domains helped identify gaps in quality-of-life research but were challenging to categorize, as classification relied on interpretive alignment with WHO guidelines and team consensus. The lack of standardized intervention protocols also limits conclusions about which components are most effective.

Second, quality-of-life measures were inconsistent and often incomplete. Only one study assessed overall quality of life (SF-36V), while others focused mainly on psychological or age-specific domains. More holistic assessments integrating physical, psychological, and social aspects are needed. Evidence certainty was generally low, highlighting the need for more rigorous designs. Additionally, studies on cognitive outcomes were unintentionally excluded, despite increasing evidence linking hearing loss to cognitive decline. Future research should incorporate cognitive function to capture broader impacts.

Despite these limitations, this review has several strengths. First, it includes a wide range of studies spanning over 30 years (1996–2024) encompassing global research from around the world, offering a robust dataset for assessing the impact of various interventions. Second, the review highlights the need for a more comprehensive approach to quality-of-life assessment, exploring multiple domains beyond psychological aspects. The inclusion of diverse interventions also provides valuable insights into potential strategies to increase the quality of life of older adults with hearing loss.

## Conclusions

In conclusion, this review provides a comprehensive synthesis and examination of the potential impact of additional interventions on the quality of life of older adults using hearing aids. The findings highlight the potential benefits and limitations of combining hearing aids with interventions such as individual at-home training, group-based auditory rehabilitation, empowerment strategies, and telehealth. Despite some limitations, this review represents the first systematic assessment of the impact of combining additional interventions with hearing aids. While further research with more rigorous designs (e.g., larger randomized controlled trials, longer follow-up periods, and standardized outcome measures) and methodologies is necessary to fully understand these effects, the value of our review lies in its synthesis of existing evidence and the presentation of findings on the basis of the studies conducted to date.

## Supplementary Information

Below is the link to the electronic supplementary material.

Supplementary Material S1: Included and Excluded Studies

Supplementary Material S2: Categorizing measurement tools on the basis of the WHOQOL-100 and WHOQOLOLD

Supplementary Material S3: Certainty of evidence

## Data Availability

All relevant data are provided within the manuscript and supplementary information files. Additional data can be obtained from the corresponding author upon reasonable request.

## Abbreviations

AIADH: Amsterdam Inventory for Auditory Disability and Handicap
ANL: Acceptable Noise Level
APHAB: Abbreviated Profile of Hearing Aid Benefit
CES-D: Center for Epidemiological Studies-Depression Scale
CID: Central Institute for the Deaf Sentence Materials
CINAHL: Cumulative Index to Nursing and Allied Health Literature
COSI: Client-Oriented Scale of Improvement
CPHI: Communication Profile for the Hearing Impaired
CST: Connected Speech Test
dB HL: Decibel Hearing Level
EMA: Ecological Momentary Assessment
EMBASE: Excerpta Medica Database
FAS: Fatigue Assessment Scale
GRADE: Grading of Recommendations, Assessment, Development, and Evaluations
HA: Hearing Aid
HASS: Hearing Aid Satisfaction Survey
HCA: Hearing Coping Assessment
HEARLI-Q: Hearing-Related Lifestyle Questionnaire
HHDI: Hearing Handicap and Disability Inventory
HHIE: Hearing Handicap Inventory for the Elderly
ICTRP: International Clinical Trials Registry Platform
IOI-AI: International Outcome Inventory for Alternative Interventions
IOI-HA: International Outcome Inventory for Hearing Aids
K-CID: Korean version of Central Institute for the Deaf test
K-HHIE: Korean version of the Hearing Handicap Inventory for the Elderly
K-IOI-HA: Korean versions of the International Outcome Inventory for Hearing Aids
MARS-HA: Measure of Audiologic Rehabilitation Self-efficacy for Hearing Aids
MEDLINE: Medical literature analysis and retrieval system online
NST: Nonsense Syllable Test
PHAP: Perception of Hearing Aid Performance
PRISMA: Preferred Reporting Items for Systematic reviews and Meta-Analyses
PROSPERO: Prospective register of systematic reviews
PsycINFO: Psychological Information database
QuickSIN: Quick Speech In Noise
RCT: Randomized Controlled Trials
RoB: Revised cochrane risk of Bias tool
SF-36V: 36-Item Short-Form health survey modified for the Veteran population
SSI: Synthetic Sentence Identification
SSQ: Speech, Spatial and Qualities of Hearing Scale
URICA-HL: University of Rhode Island Change Assessment adapted for Hearing Loss
VAS: Visual Analogue Scales
VCIT: Vowel and Consonant Imitation Test
WHOQOL: World Health Organization Quality of Life
