# Supplementary material for: Additional interventions for enhancing the quality-of-life of older adults using hearing aids: a systematic review and narrative synthesis
Source: Qual Life Res. 2026 Mar 13;35(4):98. doi: 10.1007/s11136-026-04219-7 (PMC12987892; doi:10.1007/s11136-026-04219-7)
Supplement: Supplementary file 1 — Supplementary Material S1: Included and Excluded Studies [file 11136_2026_4219_MOESM1_ESM.pdf]

Supplementary Material S1. Included and Excluded Studies

Exclusion Criteria

Reason 1 Conference abstract (n=1)

Reason 2 Protocol study (n=1)

Reason 3 Wrong study design (n=8)

Reason 4 Wrong intervention (n=12)

Reason 5 Wrong outcome (n=2)

No.	Reference	Inclusion or Exclusion (Reason)	Specific Reason for Exclusion
1	Lin, F., Albert, M. S., Alonso, A., Chisolm, T., Coker, L., Crowley, P., Deal, J., Eddins, A., Minotti, M., Mosley, T., Rebok, G., Sharrett, R., Wruck, L., & Coresh, J. (2015). Development of the achieve healthy aging study: A randomized controlled trial to determine if hearing loss treatment can reduce the risk of cognitive decline and dementia in older adults. Alzheimer’s & Dementia, 11(7), P756–P757. https://doi.org/10.1016/j.jalz.2015.06.1686	Exclusion (Reason 1)	Conference abstract
2	Deal, J. A., Albert, M. S., Arnold, M., Bangdiwala, S. I., Chisolm, T., Davis, S., Eddins, A., Glynn, N. W., Goman, A. M., Minotti, M., Mosley, T., Rebok, G. W., Reed, N., Rodgers, E., Sanchez, V., Sharrett, A. R., Coresh, J., & Lin, F. R. (2017). A randomized feasibility pilot trial of hearing treatment for reducing cognitive decline: Results from the Aging and Cognitive Health Evaluation in Elders Pilot Study. Alzheimer’s & Dementia : Translational Research & Clinical Interventions, 3(3), 410–415. https://doi.org/10.1016/j.trci.2017.06.003	Exclusion (Reason 3)	Feasibility pilot trial
3	Abrams, H. B., Bock, K., & Irey, R. L. (2015). Can a remotely delivered auditory training program improve speech-in-noise understanding? American Journal of Audiology, 24(3), 333–337. https://doi.org/10.1044/2015_AJA-15-0002	Exclusion (Reason 5)	Focused on predictors of auditory training success; no QoL outcomes assessed under WHOQOL
4	Ferguson, M., Brandreth, M., Brassington, W., & Wharrad, H. (2015). Information retention and overload in first-time hearing aid users: An interactive multimedia educational solution. American Journal of Audiology, 24(3), 329–332. https://doi.org/10.1044/2015_AJA-14-0088	Exclusion (Reason 4)	Focused on knowledge improvement via educational tool only; no QoL outcome or structured additional intervention
5	Meijerink, J. F. J., Pronk, M., & Kramer, S. E. (2020). Experiences with and lessons learned from developing, implementing, and evaluating a support program for older hearing aid users and their communication partners in the hearing aid dispensing setting. American Journal of Audiology, 29(3), 638–647. https://doi.org/10.1044/2020_AJA-19-00072	Exclusion (Reason 3)	Research note
6	Rishiq, D., Rao, A., Koerner, T., & Abrams, H. (2016). Can a commercially available auditory training program improve audiovisual speech performance? American Journal of Audiology, 25(3S), 308–312. https://doi.org/10.1044/2016_AJA-16-0017	Exclusion (Reason 3)	Research note

7	Brewster, K., Choi, C. J., He, X., Kim, A. H., Golub, J. S., Brown, P. J., Liu, Y., Roose, S. P., & Rutherford, B. R. (2022). Hearing Rehabilitative Treatment for Older Adults With Comorbid Hearing Loss and Depression: Effects on Depressive Symptoms and Executive Function. The American Journal of Geriatric Psychiatry, 30(4), 448–458. https://doi.org/10.1016/j.jagp.2021.08.006	Exclusion (Reason 4)	Compared hearing aids vs sham in depressive MDD patients
8	Mulrow, C. D., Aguilar, C., Endicott, J. E., Tuley, M. R., Velez, R., Charlip, W. S., Rhodes, M. C., Hill, J. A., & DeNino, L. A. (1990). Quality-of-life changes and hearing impairment: A randomized trial. Annals of Internal Medicine, 113(3), 188–194. https://doi.org/10.7326/0003-4819-113-3-188	Exclusion (Reason 4)	Compared hearing aids to no treatment; no additive component to HA use as required by inclusion criteria.
9	Castiglione, A., Benatti, A., Velardita, C., Favaro, D., Padoan, E., Severi, D., Pagliaro, M., Bovo, R., Vallesi, A., Gabelli, C., & Martini, A. (2016). Aging, Cognitive Decline and Hearing Loss: Effects of Auditory Rehabilitation and Training with Hearing Aids and Cochlear Implants on Cognitive Function and Depression among Older Adults. Audiology & Neurotology, 21(Suppl 1), 21–28. https://doi.org/10.1159/000448350	Exclusion<br><br>(Reason 3)	Cross-sectional and Longitudinal Study
10	Andersson, G., Green, M., & Melin, L. (1997). Behavioural hearing tactics: A controlled trial of a short treatment programme. Behaviour research and therapy, 35(6), 523-530.	Inclusion	
11	Downey, R., Gagné, N., Mohanathas, N., Campos, J. L., Pichora-Fuller, K. M., Bherer, L., Lussier, M., Phillips, N. A., Wittich, W., St-Onge, N., Gagné, J. P., & Li, K. (2023). At-home computerized executive-function training to improve cognition and mobility in normal-hearing adults and older hearing aid users: a multi-centre, single-blinded randomized controlled trial. BMC Neurology, 23(1), 1–378. https://doi.org/10.1186/s12883-023-03405-1	Exclusion (Reason 2)	Protocol study
12	Malmberg, M., Lunner, T., Kähäri, K., & Andersson, G. (2017). Evaluating the short-term and long-term effects of an internet-based aural rehabilitation programme for hearing aid users in general clinical practice: a randomised controlled trial. BMJ Open, 7(5), e013047–e013047. https://doi.org/10.1136/bmjopen-2016-013047	Exclusion (Reason 4)	Control group received only reading materials without hearing aid use
13	Watson, J., Coleman, E., Jackson, C., Bell, K., Maynard, C., Hickson, L., Forster, A., Fairhurst, C., Hewitt, C., Gardner, R., Iley, K., Gailey, L., & Thyer, N. J. (2021). Randomised controlled feasibility trial of an active communication education programme plus hearing aid provision versus hearing aid provision alone (ACE To HEAR). BMJ Open, 11(4), e043364–e043364. https://doi.org/10.1136/bmjopen-2020-043364	Exclusion (Reason 3)	Feasibility trial
14	Armitage, C. J., Lees, D., Lewis, K., & Munro, K. J. (2017). Preliminary support for a brief psychological intervention to improve first-time hearing aid use among adults. British Journal of Health Psychology, 22(4), 686-700. https://doi.org/10.1111/bjhp.12244	Inclusion	

15	Cherry, R., & Rubinstein, A. (1994). The Effect of Telephone Intervention on Success with Amplification. Ear & Hearing, 15(3), 256-261.	Inclusion	
16	Chisolm, T. H., Abrams, H. B., & McArdle, R. (2004). Short- and Long-Term Outcomes of Adult Audiological Rehabilitation. Ear and Hearing, 25(5), 464–477. https://doi.org/10.1097/01.aud.0000145114.24651.4e	Exclusion (Reason 3)	Not RCT study
17	Feenstra-Kikken, V., Van de Ven, S., Lissenberg-Witte, B. I., Pronk, M., Smits, C., Timmer, B. H. B., Polleunis, C., Besser, J., & Kramer, S. E. (2024). Effectiveness of the HEAR-Aware App for Adults Not Ready for Hearing Aids, but Open to Self-Management Support: Results of a Randomized Controlled Trial. Ear and Hearing, 45(6), 1502–1516. https://doi.org/10.1097/AUD.0000000000001533	Exclusion (Reason 4)	Participants did not use hearing aids; intervention targeted pre-hearing-aid self-management.
18	Henshaw, H., Heinrich, A., Tittle, A., & Ferguson, M. (2022). Cogmed Training Does Not Generalize to Real-World Benefits for Adult Hearing Aid Users: Results of a Blinded, Active-Controlled Randomized Trial. Ear and Hearing, 43(3), 741–763. https://doi.org/10.1097/AUD.0000000000001096	Exclusion (Reason 4)	Both the intervention and control groups received similar auditory-cognitive training programs rather than comparing hearing aids alone versus hearing aids plus an additional structured intervention.
19	Hickson, L., Worrall, L., & Scarinci, N. (2007). A Randomized Controlled Trial Evaluating the Active Communication Education Program for Older People with Hearing Impairment. Ear and Hearing, 28(2), 212–230. https://doi.org/10.1097/AUD.0b013e31803126c8	Exclusion (Reason 4)	No group received hearing aid alone.
20	Humes, L. E., Skinner, K. G., Kinney, D. L., Rogers, S. E., Main, A. K., & Quigley, T. M. (2019). Clinical Effectiveness of an At-Home Auditory Training Program: A Randomized Controlled Trial. Ear & Hearing, 40(5), 1043-1060. https://doi.org/10.1097/AUD.0000000000000688	Inclusion	
21	Saunders, G. H., Smith, S. L., Chisolm, T. H., Frederick, M. T., McArdle, R. A., & Wilson, R. H. (2016). A Randomized Control Trial: Supplementing Hearing Aid Use with Listening and Communication Enhancement (LACE) Auditory Training. Ear and Hearing, 37(4), 381–396. https://doi.org/10.1097/AUD.0000000000000283	Exclusion (Reason 4)	Control groups also used HAs and received counseling or placebo training; lacked HA-only comparison group.
22	Vreeken, H. L., van Nispen, R. M. A., Kramer, S. E., & van Rens, G. H. M. B. (2020). 'Dual Sensory Loss Protocol' for Communication and Wellbeing of Older Adults With Vision and Hearing Impairment – A Randomized Controlled Trial. Frontiers in Psychology, 11(570339). https://doi.org/10.3389/fpsyg.2020.570339	Inclusion	
23	Yu, J., Jeon, H., Song, C., & Han, W. (2017). Speech perception enhancement in elderly hearing aid users using an auditory training program for mobile devices. Geriatrics & Gerontology International, 17(1), 61–68. https://doi.org/10.1111/ggi.12678	Exclusion (Reason 4)	Group education program without comparison to hearing-aid-only group

24	Kramer, S. E., Allessie, G. H. M., Dondorp, A. W., Zekveld, A. A., & Kapteyn, T. S. (2005). A home education program for older adults with hearing impairment and their significant others: A randomized trial evaluating short- and long-term effects. International Journal of Audiology, 44(5), 255-264. https://doi.org/10.1080/14992020500060453	Inclusion	
25	Lelic, D., Parker, D., Herrlin, P., Wolters, F., & Smeds, K. (2024). Focusing on positive listening experiences improves hearing aid outcomes in experienced hearing aid users. International journal of audiology, 63(6), 420-430.	Inclusion	
26	Rey, G., Knoblauch, K., Jouvent, R., Collet, L., & Dubal, S. (2010). The experience of pleasure before and after hearing rehabilitation. International Journal of Rehabilitation Research, 33(2), 158–164. https://doi.org/10.1097/MRR.0b013e328331c5c8	Exclusion (Reason 3)	Not RCT study
27	Han, J. S., Lim, J. H., Kim, Y., Aliyeva, A., Seo, J.-H., Lee, J., & Park, S. N. (2024). Hearing rehabilitation with a chat-based mobile auditory training program in experienced hearing aid users: Prospective randomized controlled study. JMIR mHealth and uHealth, 12, e50292. https://doi.org/10.2196/50292	Inclusion	
28	Meijerink, J. F., Pronk, M., Lissenberg-Witte, B. I., Jansen, V., & Kramer, S. E. (2020). Effectiveness of a web-based SUpport PRogram (SUPR) for hearing aid users aged 50+: two-arm, cluster randomized controlled trial. Journal of medical Internet research, 22(9), e17927.	Inclusion	
29	Abrams, H., Chisolm, T. H., & McArdle, R. (2002). A cost-utility analysis of adult group audiologic rehabilitation: Are the benefits worth the cost? Journal of Rehabilitation Research and Development, 39(5), 549-558.	Inclusion	
30	Stecker, G. C., Bowman, G. A., Yund, E. W., Herron, T. J., Roup, C. M., & Woods, D. L. (2006). Perceptual training improves syllable identification in new and experienced hearing aid users. Journal of Rehabilitation Research & Development, 43(4), 537-552. https://doi.org/10.1682/JRRD.2005.11.0171	Inclusion	
31	Smith, S. L., Saunders, G. H., Chisolm, T. H., Frederick, M., & Bailey, B. A. (2016). Examination of individual differences in outcomes from a randomized controlled clinical trial comparing formal and informal individual auditory training programs. Journal of Speech, Language, and Hearing Research, 59(4), 876–886. https://doi.org/10.1044/2016_JSLHR-H-15-0162	Exclusion (Reason 5)	Evaluated training delivery modes and predictors rather than additive intervention effect on QoL.
32	Lundberg, M., Andersson, G., & Lunner, T. (2011). A Randomized, Controlled Trial of the Short-Term Effects of Complementing an Educational Program for Hearing Aid Users with Telephone Consultations. Journal of the American Academy of Audiology, 22(10), 654–662. https://doi.org/10.3766/jaaa.22.10.4	Exclusion (Reason 4)	Both groups used hearing aids and received some educational material; intervention not clearly distinct.
33	Olson, A. D., Preminger, J. E., & Shinn, J. B. (2013). The Effect of LACE DVD Training in New and Experienced Hearing Aid Users. Journal of the American Academy of Audiology, 24(3), 214-230. https://doi.org/10.3766/jaaa.24.3.7	Inclusion	

34	Sweetow, R. W., & Sabes, J. H. (2006). The Need for and Development of an Adaptive Listening and Communication Enhancement (LACETM) Program. Journal of the American Academy of Audiology, 17(8), 538–558. https://doi.org/10.3766/jaaa.17.8.2	Exclusion (Reason 3)	Not RCT study
35	Thorén, E., Svensson, M., Törnqvist, A., Andersson, G., Carlbring, P., & Lunner, T. (2011). Rehabilitative Online Education versus Internet Discussion Group for Hearing Aid Users: A Randomized Controlled Trial. Journal of the American Academy of Audiology, 22(5), 274–285. https://doi.org/10.3766/jaaa.22.5.4	Exclusion (Reason 4)	No HA-only control group; both groups received active interventions with hearing aids.
36	Lin, F. R., Pike, J. R., Albert, M. S., Arnold, M., Burgard, S., Chisolm, T., Couper, D., Deal, J. A., Goman, A. M., Glynn, N. W., Gmelin, T., Gravens-Mueller, L., Hayden, K. M., Huang, A. R., Knopman, D., Mitchell, C. M., Mosley, T., Pankow, J. S., Reed, N. S., … Coresh, J. (2023). Hearing intervention versus health education control to reduce cognitive decline in older adults with hearing loss in the USA (ACHIEVE): a multicentre, randomised controlled trial. The Lancet (British Edition), 402(10404), 786–797. https://doi.org/10.1016/S0140-6736(23)01406-X	Exclusion (Reason 4)	Control group: Health education program focused on healthy aging (no hearing aid)
