# Supplementary material for: Additional interventions for enhancing the quality-of-life of older adults using hearing aids: a systematic review and narrative synthesis
Source: Qual Life Res. 2026 Mar 13;35(4):98. doi: 10.1007/s11136-026-04219-7 (PMC12987892; doi:10.1007/s11136-026-04219-7)
Supplement: Supplementary file 2 — Supplementary Material S2: Categorizing measurement tools on the basis of the WHOQOL-100 and WHOQOLOLD [file 11136_2026_4219_MOESM2_ESM.pdf]

Supplementary Material S2. Categorizing measurement tools on the basis of the WHOQOL-100 and WHOQOL-OLD (Power et al., 2005; WHO, 1998b)

Domain	Facet	Outcomes related to QoL	Measurement tools
Overall quality of life		Quality of life	SF-36V
Physical	Energy and fatigue	Severity of fatigue	FAS
Psychological	Positive feelings	Satisfaction with HA	APHAB, Ad hoc research tool, HASS, K-IOI-HA, APHAB
		Benefit, Residual activity limitation, Satisfaction, Residual participation restriction, Impact on others, Quality of life, Effectiveness of HA treatment	IOI-HA, IOI-AI
		Hearing-related lifestyle	HEARLI-Q
		Momentary HA satisfaction	EMA
	Negative feelings	Anxiety about ageing	Lasher and Faulkender's anxiety about aging scale
		Number of complaints using HA	Ad hoc research tool
		Perceived hearing handicap	HHIE, K-HHIE, AIADH
		Depressive symptoms	CES-D
		Feeling of loneliness	De Jong Gierveld Loneliness Scale
		Emotional response and communication strategies	HHDI
	Self-esteem	Motivation to use HA	Ad hoc research tool
		Self-efficacy for HA handling	MARS-HA
		Readiness to act on hearing loss	URICA-HL
Older-adult-specific	Sensory abilities	Hearing problems	VAS
		Speech recognition in noise	CST, NHT, QuickSIN
		Speech understanding	Compressed speech test, SSI, SSQ
		The direct impact of training	Trained frequent-word speech materials
		Participant perception of hearing aid performance	PHAP
		Tolerance of background noise while listening to speech	ANL

		Final ability in the situations that participants would like to improve<br>Phoneme detection<br>Phoneme perception<br>Word perception ability<br><br>Sentence perception ability<br>Syllable identification in noise	COSI for "final ability"<br><br>Ling Six Sound test<br>VCIT<br>Monosyllable and Bisyllable open-set tests<br>CID, K-CID<br>NST
	Autonomy	Communication strategies, Personal adjustment to hearing loss<br>Coping behavior<br>Degree of change in the situations that participants would like to improve	CPHI<br><br>HCA<br>COSI for "degree of change"

Abbreviations: AIADH, Amsterdam Inventory for Auditory Disability and Handicap; ANL, Acceptable Noise Level; APHAB, Abbreviated Profile of Hearing Aid Benefit; CES-D, Center for Epidemiological Studies – Depression Scale; CID, Central Institute for the Deaf sentence materials; COSI, Client-Oriented Scale of Improvement; CPHI, Communication Profile for the Hearing Impaired; CST, Connected Speech Test; dB HL, decibels hearing level; EMA, Ecological Momentary Assessment; FAS, Fatigue Assessment Scale; HA, Hearing Aid; HASS, Hearing Aid Satisfaction Survey; HCA, Hearing Coping Assessment; HEARLI-Q, Hearing-Related Lifestyle Questionnaire; HHDI, Hearing Handicap and Disability Inventory; HHIE, Hearing Handicap Inventory for the Elderly; IOI-AI, International Outcome Inventory for Alternative Interventions; IOI-HA, International Outcome Inventory for Hearing Aids; K-CID, Korean version of Central Institute for the Deaf test; K-HHIE, Korean version of the Hearing Handicap Inventory for the Elderly; K-IOI-HA, Korean versions of the International Outcome Inventory for Hearing Aids; MARS-HA, Measure of Audiologic Rehabilitation Self-Efficacy for Hearing Aids; NST, Nonsense Syllable Test; PHAP, Perception of Hearing Aid Performance; QuickSIN, Quick Speech in Noise; SF-36V, 36-Item Short-Form Health Survey modified for the Veteran population; SSI, Synthetic Sentence Identification; SSQ, Speech, Spatial and Qualities of Hearing Scale; URICA-HL, University of Rhode Island Change Assessment adapted for Hearing Loss; VAS, Visual Analogue Scales; VCIT, Vowel and Consonant Imitation Test

1. Physical: This domain includes the assessment of a physical facet of quality of life which is energy and fatigue.

1.1 Energy and fatigue facet (i.e., fatigue) was measured using the Fatigue Assessment Scale (FAS) (Michielsen et al., 2003; Michielsen et al., 2004).

2. Psychological: This domain includes the assessment of psychological facets of quality of life which include positive or negative feelings, and self-esteem.

2.1 Positive feelings facet (e.g., satisfaction with hearing aid use) was measured using the Abbreviated Profile of Hearing Aid Benefit (APHAB) (Yun et al., 2000), the Ecological Momentary Assessment (EMA) (Stone et al., 2007), the Hearing Aid Satisfaction Survey (HASS) (Kochkin, 1997), the International Outcomes Inventory for Alternative Interventions (IOI-AI) (Noble, 2002), the International Outcomes Inventory for Hearing Aids (IOI-HA) (Cox & Alexander, 2002), the Korean versions of the International Outcome Inventory for Hearing Aids (K-IOI-HA) (Chu et al., 2012), the Satisfaction with Amplification in Daily Life (SADL) (Cox & Alexander, 1999), and the self-

developed tool created for the study (Armitage et al., 2017; Cherry & Rubinstein, 1994; Kramer et al., 2005; Lelic et al., 2024).

2.2 Negative feelings facet (e.g., anxiety, depression, loneliness, discomfort) was measured using the Amsterdam Inventory for Auditory Disability and Handicap (AIADH) (Kramer et al., 1995; Meijer et al., 2003), the Center for Epidemiological Studies - Depression Scale (CES-D) (Radloff, 1977), the De Jong Gierveld Loneliness Scale (De Jong-Gierveld & Van Tilburg, 1999), the Glasgow Hearing Aid Benefit Profile (GHABP) (Gatehouse, 1999), the Hearing Handicap and Disability Inventory (HHDI) (Brink, 1995; Kramer et al., 2005), the Hearing Handicap Inventory for the Elderly (HHIE) (Ventry & Weinstein, 1982), the Hospital Anxiety and Depression Scale (HADS) (Zigmond & Snaith, 1983), the Korean version of the Hearing Handicap Inventory for the Elderly (K-HHIE) (Park et al., 2011), the Lasher and Faulkender's Anxiety About Aging Scale (Lasher & Faulkender, 1993), as well as daily registrations of hearing problems (Andersson et al., 1997) and counting the number of complaints (Cherry & Rubinstein, 1994).

2.3 Self-esteem facet (e.g., self-efficacy in the use of hearing aids and readiness to address hearing loss) was measured using the Measure of Audiologic Rehabilitation Self-efficacy for Hearing Aids (MARS-HA) (West & Smith, 2007), and the University of Rhode Island Change Assessment adapted for Hearing Loss (URICA-HL) (Pronk et al., 2017).

3. Older-adult-specific: This domain includes the assessment of older-adult-specific facets of quality of life which include sensory abilities, and autonomy.

3.1 Sensory abilities facet (e.g., speech recognition, sentence recognition, perception of hearing aid performance, hearing ability) was measured using the Abbreviated Profile of Hearing Aid Performance (APHAP) (Cox & Alexander, 1995), the Acceptable Noise Level (ANL) (Nabelek et al., 2006), the Central Institute for the Deaf (CID) test (Davis & Silverman, 1978), the Client-Oriented Scale of Improvement (COSI) - degree of change (Dillon et al., 1997), the Compressed Speech Test (Department of Veterans Affairs, 1998), the Connected Speech test (CST) (Cox et al., 1987), the Korean version of Central Institute for the Deaf (K-CID) test (JANG et al., 2012), the Ling Six Sound test (Glista et al., 2014), the Monosyllable and Bisyllable open-set test (Lee et al., 2010), the Nonsense Syllable Test (NST) (Dubno & Levitt, 1981), the Participant perception of Hearing Aid Performance (PHAP) (Cox & Gilmore, 1990), the Quick Speech in Noise (QuickSIN) (Killion et al., 2004), the Speech, Spatial and Qualities of Hearing Scale (SSQ) (Gatehouse & Noble, 2004), the Synthetic Sentence Identification (SSI) (Speaks & Jerger, 1965), the Visual Analogue Scale (VAS) (Andersson et al., 1997), and the Vowel and Consonant Imitation Test (VCIT) (KIM et al., 1997).

3.2 Autonomy facet (e.g., communication and coping strategies, coping abilities) was measured using the Client-Oriented Scale of Improvement (COSI) - final ability (Dillon et al., 1997), the Communication Strategies Subscales of the Communication Profile for the Hearing Impaired (CPHI) (Demorest & Erdman, 1987), the Hearing Coping Assessment (HCA) (Andersson et al., 1995), and the Self-Assessment of Communication (SAC) (Schow & Nerbonne, 1982).
