# Supplementary material for: Additional interventions for enhancing the quality-of-life of older adults using hearing aids: a systematic review and narrative synthesis
Source: Qual Life Res. 2026 Mar 13;35(4):98. doi: 10.1007/s11136-026-04219-7 (PMC12987892; doi:10.1007/s11136-026-04219-7)
Supplement: Supplementary file 3 — Supplementary Material S3: Certainty of evidence [file 11136_2026_4219_MOESM3_ESM.pdf]

Supplementary Material S3. Certainty of evidence

**Outcomes** [a]	**Number of participants (studies)**	**Findings**	**Certainty of evidence (GRADE)**
Overall quality of life	105 (1 RCT)	No statistically significant differential treatment effects.	Very low ⊕○○○
Physical domain	131 (1 RCT)	No statistically significant differential treatment effects.	Low ⊕⊕○○
Psychological domain	737 (9 RCT)	* Significantly less anxious about ageing and more accepting of older people than were participants in the control group (between-group difference = 0.75, 95% CI = 0.26, 1.22, d = 0.87).<br>* Significantly higher self-efficacy for advanced HA handling than the controls, which was sustained at 12 months (MARS-HA; mean difference immediately postintervention: 5.3, 95% CI 0.3 to 10.4; P=.04).<br>* Significantly greater HA satisfaction than controls immediately postintervention (IOI-HA; 0.3, 95% CI 0.09 to 0.5; P=.006), which was sustained at 12 months.<br>* Reflected on the positive experiences with HAs than control group.<br>* An improvement of positive feelings in communication strategies in the training group was observed after the treatment (postintervention), while no improvement was observed in the control group (p<0.05).<br>* While a relapse was found in the control group at 6 months follow-up, an improvement in enjoyment of life was experienced in the training group.	Low ⊕⊕○○
Older-adult-specific domain	656 (8 RCT)	* In the CPHI (Communication Strategies Subscales) test for autonomy, a significant treatment effect was found [Wilk's 2 (2,14) = 0.347, P = 0.0006].<br>* Only for the VAS dealing with daily problems with hearing was a significant result found (P<.01).<br>* In the bisyllable condition (word perception test), significantly better results compared to the control group (P=.04, linear mixed model).<br>* In the K-CID test (sentence perception test), significantly greater improvement than the control group (P=.03, linear mixed model).<br>* Better improvement in word and sentence perception tests compared to the control group (n=20; P=.04 and P=.03, respectively), while no significant difference was observed in phoneme and consonant perception tests (both P>.05).<br>* Both the degree of change and final ability were significantly higher in the intervention group.<br>* Improvement in Nonsense Syllable Test (NST) performance who underwent training and training-related improvements were significantly greater than the performance gains during the acclimatization period (Immediate Training group: $F_{1,21} = 40.5$, $p < 0.001$; Delayed Training group: $F_{1,18} = 33.4$, $p < 0.001$).	Low ⊕⊕○○

[a] Categorized by domain based on the WHOQOL-100 and WHOQOL-OLD
